# Phage biobanks as enabling infrastructure for precision phage therapy in the era of antimicrobial resistance

**DOI:** 10.3389/frabi.2026.1772871

**Published:** 2026-01-30

**Authors:** Paulina Topa-Pila, Katheryne Y. Morales, Doménica P. Palacios-Mora, Cristina Flores-Hernández, Analía Altamirano-Cisneros, Joselyn Micaela Gavilanes, Keyla Villacís-López, Mirari Arancibia, Johanna Mora-Domínguez, William Calero-Cáceres

**Affiliations:** 1 Biotechnology Program, Department of Food and Biotechnology Science and Engineering, Universidad Técnica de Ambato, Ambato, Ecuador; 2 Clínica Santa Ana, Cuenca, Ecuador; 3 Departamento de Ciencias Exactas Sede Latacunga, Universidad de las Fuerzas Armadas ESPE, Rumiñahui, Ecuador; 4 Hospital de Especialidades José Carrasco Arteaga, Instituto Ecuatoriano de Seguridad Social (IESS), Cuenca, Ecuador; 5 UTA-RAM-One Health, Group for Universal Advance in Bioscience, Department of Food and Biotechnology Science and Engineering, Universidad Técnica de Ambato, Ambato, Ecuador

**Keywords:** antimicrobial resistance (AMR), bioinformatics, One health, phage biobanks, phage therapy, precision medicine

## Abstract

The renewed interest in bacteriophage therapy as a response to antimicrobial resistance (AMR) has exposed a critical barrier to its scalable implementation: the lack of structured infrastructure to support precision phage deployment. Due to their narrow host specificity, bacteriophages require timely access to well-matched candidates, making *ad hoc* isolation and informal exchange insufficient for routine therapeutic use. In this Perspective, we argue that phage biobanks must be redefined as enabling infrastructure rather than passive repositories. We propose that next-generation phage biobanks should integrate curated biological diversity, systematic genomic and functional qualification, and predictive capacity to support rapid phage–host matching. Together, these elements transform biobanks into decision-support systems capable of informing translational applications under time-sensitive conditions. This infrastructural model is particularly relevant within a One Health framework, where resistant pathogens circulate across human, veterinary, agricultural, and environmental domains. Rather than reviewing methodologies, we outline conceptual principles to guide the design of phage biobanks as integrated, predictive, and sustainable assets. We contend that the future impact of phage therapy will depend less on individual phage discovery than on the development of interoperable biobank infrastructures that enable precision antimicrobial interventions at scale.

## 1 Introduction – why infrastructure, not collections

The global escalation of antimicrobial resistance (AMR) has reopened the therapeutic landscape to alternatives and complementary strategies beyond conventional antibiotics, placing bacteriophages back into focus as precision antibacterial agents that can be deployed alone or in combination with existing antimicrobial therapies (Garvey, 2020). Unlike small-molecule antimicrobials, bacteriophages exhibit a high degree of host specificity, often acting at the level of bacterial species, subspecies, or even individual strains (Peng et al., 2025). This biological property, while advantageous in terms of selectivity and microbiome preservation, introduces a fundamental structural challenge for the deployment of phage therapy at scale: effective treatment depends on timely access to phages that match the infecting bacterial isolate.

To date, much of clinical and experimental phage therapy has relied on *ad hoc* strategies, including bespoke phage isolation, informal inter-laboratory exchanges, or emergency compassionate-use networks (Jones et al., 2023; Niazi, 2025). Although these approaches have enabled remarkable individual successes, they remain inherently reactive, fragmented, and poorly scalable. In this context, the absence of organized, interoperable phage biobanks represents a critical bottleneck for the broader translation of phage therapy into routine clinical, veterinary, and agricultural practice (Zalewska-Piątek, 2023).

Phage biobanks are often discussed as repositories of biological material; however, such a definition underestimates their strategic role. In the AMR era, phage biobanks should be understood as enabling infrastructure: systems designed to support rapid decision-making, quality assurance, and translational deployment of phage therapeutics (Lin et al., 2021). Their function extends beyond storage to include the curation of biological diversity, the integration of genomic and functional information, and the facilitation of phage–host matching under time-sensitive conditions. This infrastructural perspective is particularly relevant in a One Health framework, where resistant bacterial pathogens circulate across human, animal, agricultural, and environmental interfaces (Samson et al., 2024). A biobank capable of supporting precision phage therapy in one sector can, if properly designed, serve multiple domains simultaneously. Thus, phage biobanks occupy a unique position at the intersection of microbiology, data science, and translational medicine.

In this Perspective, we argue that the future success of phage therapy depends not primarily on the discovery of individual “ideal” phages, but on the development of phage biobanks as integrated, predictive, and interoperable infrastructure. Rather than providing an exhaustive review of methodologies, we focus on the conceptual principles that should guide the design and function of next-generation phage biobanks, positioning them as foundational tools for precision antimicrobial therapy.

## 2 Host specificity as the architectural constraint

The defining biological feature of bacteriophages (their narrow host specificity) constitutes the primary architectural constraint shaping the design of phage biobanks. Unlike antibiotics, whose activity often spans multiple species or genera, most phages infect only a limited subset of bacterial strains. This specificity underpins the precision of phage therapy but simultaneously precludes universal or “one-size-fits-all” therapeutic solutions (Jones et al., 2023).

From an infrastructural standpoint, host specificity transforms phage therapy from a problem of *drug availability* into a problem of *matching*. Effective deployment depends not on the existence of a single highly potent phage, but on access to a sufficiently diverse repertoire capable of covering the genetic and phenotypic heterogeneity of target bacterial populations (Strathdee et al., 2023). Consequently, phage biobanks must be designed around diversity, redundancy, and continuous expansion, rather than around a fixed or finite collection.

This constraint has direct implications for how biobanks define their scope. Species-level representation is insufficient; clinically and environmentally relevant variation often occurs at the strain level, driven by differences in surface receptors, mobile genetic elements, and resistance mechanisms (Egido et al., 2022). A biobank optimized for precision therapy must therefore prioritize depth within high-priority bacterial taxa, rather than breadth across unrelated organisms (Kaur et al., 2021). In this sense, the value of a phage biobank is not measured by the absolute number of phages it contains, but by how effectively its collection maps onto the diversity of circulating bacterial hosts.

Host specificity also imposes temporal constraints. Bacterial populations evolve rapidly, particularly under antimicrobial pressure, leading to shifts in susceptibility profiles and receptor usage (Zhao et al., 2024). Static collections risk obsolescence unless they are continuously refreshed and re-evaluated. Phage biobanks must therefore be conceived as dynamic infrastructures, capable of adapting to changing bacterial landscapes rather than preserving historical snapshots of phage diversity (Resch et al., 2024). Importantly, host specificity should not be viewed as a limitation to overcome, but as a design principle to embrace. When properly leveraged, it enables targeted interventions that minimize off-target effects and preserve beneficial microbiota (Cui et al., 2024). However, realizing this advantage at scale requires biobanks that are explicitly structured to accommodate specificity through strategic collection growth, curated host coverage, and integration with downstream qualification and matching processes. In this framework, host specificity is not merely a biological characteristic of phages; it is the organizing logic around which effective phage biobanks must be built.

## 3 From isolation to qualification: making phages “bankable”

The transition from individual phage isolates to functional elements of a biobank requires a conceptual shift from discovery to qualification. In the context of precision phage therapy, not every isolated phage is suitable for inclusion in a biobank. Instead, phages must undergo a process of biological, informational, and externally anchored validation that renders them bankable: safe, interpretable, and deployable within a therapeutic pipeline (Panagiotopoulos et al., 2025). This qualification process extends beyond internal laboratory characterization and increasingly aligns with independent quality frameworks, including those articulated in the European Pharmacopoeia General Chapter 5.31 for phage therapy medicinal products, which define reference expectations for identity, genomic integrity, purity, and safety (European Directorate for the Quality of Medicines & HealthCare of the Council of Europe (EDQM), 2024). In this sense, “bankability” reflects not only scientific suitability, but compatibility with externally recognizable standards that support translational use.

At this stage, isolation alone is insufficient. While environmental and clinical sampling continue to expand the known diversity of bacteriophages, the translational value of these discoveries depends on their systematic qualification. Phage biobanks therefore operate not as passive repositories of isolates, but as curated systems that apply defined inclusion criteria (Xing et al., 2025). Central to this process is genomic qualification, which serves as the primary gatekeeper for biobank entry (Calero-Cáceres and Balcázar, 2025). Whole-genome analysis allows the exclusion of phages carrying genes associated with lysogeny, virulence, or horizontal gene transfer, thereby aligning biobank contents with safety expectations for therapeutic use (Gholizadeh et al., 2024). Beyond safety, genomic characterization provides a stable reference framework for classification, traceability, and interoperability. In contrast to phenotypic assays that may vary with experimental conditions, genomic data constitute a reproducible and transferable layer of information that anchors each phage within the biobank (Banerjee et al., 2025). This genomic anchoring is essential for regulatory dialogue, inter-institutional exchange, and long-term collection management.

Functional qualification complements genomic screening by establishing the biological relevance of a phage within its intended context of use. Rather than exhaustive phenotyping, biobanks must define functional criteria that are directly aligned with translational objectives, such as demonstrable lytic activity against representative host strains or efficacy under conditions relevant to infection biology (Lin et al., 2021). Importantly, this functional layer is not designed to predict therapeutic outcomes in isolation, but to ensure that phages included in the biobank possess a minimal and interpretable activity profile (Zalewska-Piątek, 2023). Crucially, qualification is not a one-time event. As bacterial populations evolve and new genomic insights emerge, phages may require periodic re-evaluation. This reinforces the notion of phage biobanks as dynamic infrastructures rather than static archives. Continuous curation, re-annotation, and contextual updating are integral to maintaining the relevance of the collection over time.

By formalizing the transition from isolation to qualification, phage biobanks establish a common language between microbiology, genomics, and translational application (Resch et al., 2024). In doing so, they create the conditions necessary for downstream integration with predictive tools and decision-support systems, positioning qualified phages not merely as biological entities, but as informed therapeutic assets.

## 4 Predictive phage biobanks: from catalogs to decision engines

Once phages are qualified and curated within a biobank, their value is no longer defined solely by their biological properties, but by how effectively they can inform therapeutic decisions. In this context, the role of phage biobanks evolves from that of catalogs of biological material to decision-support infrastructures capable of guiding phage selection under time-sensitive conditions. This transition marks a critical inflection point for the scalability of precision phage therapy.

Predictive capacity in phage biobanks does not rely on a single computational approach, but on the integration of multiple layers of information generated during the qualification process. Genomic data, host-range profiles, and contextual metadata together create a structured knowledge base that can be interrogated to infer likely phage–host compatibility (Baláž et al., 2023). Rather than replacing experimental validation, predictive frameworks serve to narrow the solution space, enabling faster and more rational prioritization of candidate phages (Wu et al., 2025). *In silico* approaches are particularly valuable in addressing the combinatorial complexity imposed by host specificity. As biobanks expand to encompass hundreds or thousands of phages and an equally diverse array of bacterial hosts, exhaustive empirical screening becomes increasingly impractical. Computational inference (drawing on genomic signatures, comparative analyses, and accumulated interaction data) offers a means to triage candidates efficiently while preserving biological interpretability (Nie et al., 2024). An important conceptual development in this context is the notion of standardized phage metadata, which could be described as “phage passports” (Green, 2024). These structured descriptors consolidate essential information, including genomic identity, safety status, functional qualification, and known host associations. By harmonizing how phages are described and queried, phage passports could enable interoperability across biobanks and facilitate the integration of predictive tools without imposing methodological uniformity.

**Figure 1:**
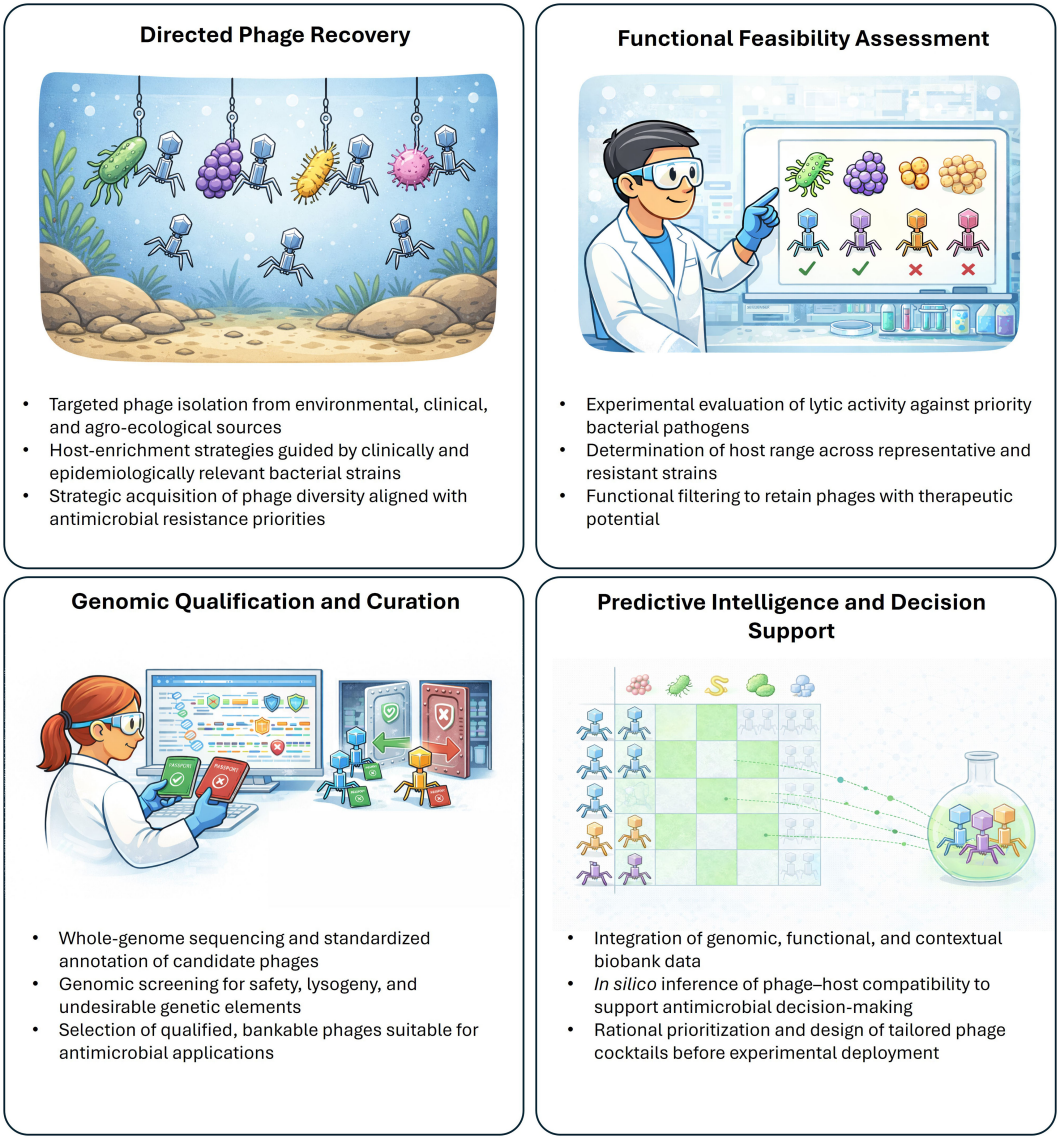
Conceptual architecture of a predictive phage biobank supporting precision antimicrobial applications.

Crucially, the predictive capacity of phage biobanks is fundamentally constrained by methodological heterogeneity across both *wet-lab* and *in silico* workflows. Reliable prediction of phage–host interactions, lytic potential, or therapeutic suitability depends on the availability of high-quality, comparable phage and bacterial genomes; however, no harmonized standards currently exist for phage DNA extraction, library preparation, sequencing strategies, genome assembly, or annotation (Mora-Domínguez and Calero-Cáceres, 2025). Variability at any of these stages propagates into downstream analyses, limiting the interpretability and transferability of predictive models (Jansz and Faulkner, 2024). In parallel, computational approaches for host-range inference and functional annotation remain under active development, with a substantial fraction of phage coding sequences still lacking confident functional assignments. Accordingly, prediction should be viewed not as a stand-alone computational solution, but as an emergent property of systematically qualified, coherently annotated, and methodologically standardized biobank datasets. These considerations underscore that the transition from catalogs to truly predictive phage biobanks requires deliberate harmonization of experimental and analytical pipelines, thereby setting the stage for broader translational integration and governance considerations. The functional organization of a predictive phage biobank —integrating targeted recovery, functional feasibility assessment, genomic qualification, and *in silico* decision support— is summarized in **Figure 1**.

## 5 Translational integration across one health

The translational value of phage biobanks extends beyond any single sector, reflecting the ecological reality that antimicrobial resistance circulates across human, animal, agricultural, and environmental interfaces. From a One Health perspective, the relevance of phage biobanks is not defined by sector-specific applications, but by their ability to support precision interventions across interconnected systems (Jo et al., 2023). This integration emerges not as an added conceptual layer, but as a direct consequence of infrastructural design capable of operating across biological and institutional boundaries.

Across domains, the functional requirements for phage deployment are largely shared. Whether applied to multidrug-resistant infections in clinical settings, bacterial diseases in livestock, or pathogen control in agricultural systems, effective phage use depends on rapid access to qualified phages, reliable host matching, and contextual interpretation of activity (Faruk et al., 2025). Differences between sectors primarily concern deployment tempo and regulatory context rather than underlying biological principles: clinical applications often require rapid, case-specific responses, whereas veterinary and agricultural contexts may prioritize preventive or population-level strategies (Strathdee et al., 2023; Marino et al., 2025). A single biobank infrastructure can accommodate these divergent needs by supporting multiple operational modes, provided that qualification standards and data structures remain interoperable.

Importantly, many priority bacterial pathogens traverse sectoral boundaries, including *Escherichia coli*, *Salmonella enterica*, *Staphylococcus aureus*, and *Enterococcus* spp., frequently carrying shared resistance determinants (Birlutiu and Birlutiu, 2025; Sati et al., 2025). Phage biobanks structured around such taxa can therefore generate cross-sector benefits, enabling reuse of qualified phages and associated knowledge without duplicating discovery efforts. Beyond direct intervention, this integrative capacity also supports surveillance and preparedness by linking phage collections to evolving bacterial landscapes. In this sense, the principal challenge for One Health implementation is not phage applicability, but infrastructural fragmentation. Interoperable phage biobanks offer a shared backbone for discovery, qualification, and deployment, enabling coordinated responses to antimicrobial resistance that reflect the biological continuity of the problem itself.

## 6 Governance, standardization, and the future of phage biobank infrastructure

The capacity of phage biobanks to function as effective infrastructure depends not only on scientific rigor, but also on governance and standardization frameworks that enable interoperability, scalability, and long-term viability. In the absence of such enabling structures, even well-curated collections risk fragmentation, duplication of effort, and limited translational impact (Bledsoe and Watson, 2025; Mayrhofer, 2025). Governance and standardization should therefore be viewed not as ancillary considerations, but as foundational conditions that allow phage biobanks to evolve from isolated initiatives into coordinated systems supporting precision antimicrobial interventions (Mutalik and Arkin, 2022).

Standardization is central to this transition, particularly with respect to phage qualification, conservation procedures, purification workflows, genomic annotation, and minimal functional metadata (Luong et al., 2020; Turner et al., 2021; Mora-Domínguez and Calero-Cáceres, 2025). Harmonized descriptors enable phages to be meaningfully compared, exchanged, and deployed across institutions without imposing methodological uniformity. Rather than enforcing identical protocols, standardization establishes shared reference points that allow diverse experimental and analytical pipelines to converge into interoperable outputs, forming the basis for predictive tools, regulatory dialogue, and cross-institutional collaboration.

Looking forward, the long-term impact of phage biobanks will depend on their ability to operate as sustained, coordinated infrastructure embedded within public-health and research ecosystems. Interoperable biobank networks, supported by hub-and-spoke governance models, can accommodate local needs while maintaining coherence across clinical, veterinary, and agricultural domains. Recognizing phage biobanks as public-good infrastructure (rather than short-lived research projects) provides a rationale for sustained institutional support and integration with surveillance and diagnostic pipelines. In this sense, phage biobanks form the backbone of future phage therapeutic ecosystems, enabling the field to move beyond proof-of-concept applications toward a scalable and resilient response to antimicrobial resistance.

## References

[B1] Baláž A. Kajsik M. Budiš J. Szemes T. Turňa J. (2023). PHERI—Phage host exploRation pipeline. Microorganisms 11, 1398. doi: 10.3390/MICROORGANISMS11061398/S1 37374901 PMC10305434

[B2] Banerjee B. Halder S. Kumar S. Chaddha M. Ali R. Mohite R. . (2025). Genomic insights into bacteriophages: a new frontier in AMR detection and phage therapy. Brief Funct. Genomics 24, elaf011. doi: 10.1093/BFGP/ELAF011, PMID: 40720171 PMC12302716

[B3] Birlutiu V. Birlutiu R. M. (2025). An overview of the epidemiology of multidrug resistance and bacterial resistance mechanisms: what solutions are available? A comprehensive review. Microorganisms 13 (9), 2194. doi: 10.3390/microorganisms13092194, PMID: 41011524 PMC12472688

[B4] Bledsoe M. J. Watson P. H. (2025). Biobanks—Organization, operations, and role in genomics research. Genomics Populations Soc., 85–120. doi: 10.1016/B978-0-323-91799-5.00003-6

[B5] Calero-Cáceres W. Balcázar J. L. (2025). Progress in phage therapy in response to antimicrobial resistance. Virology 609, 110578. doi: 10.1016/j.virol.2025.110578, PMID: 40410050

[B6] Cui L. Watanabe S. Miyanaga K. Kiga K. Sasahara T. Aiba Y. . (2024). A comprehensive review on phage therapy and phage-based drug development. Antibiotics 13 (9), 870. doi: 10.3390/antibiotics13090870, PMID: 39335043 PMC11428490

[B7] Egido J. E. Costa A. R. Aparicio-Maldonado C. Haas P. J. Brouns S. J. J. (2022). Mechanisms and clinical importance of bacteriophage resistance. FEMS Microbiol. Rev. 46 (1), fuab048. doi: 10.1093/femsre/fuab048, PMID: 34558600 PMC8829019

[B8] European Directorate for the Quality of Medicines & HealthCare of the Council of Europe (EDQM) (2024). European Pharmacopoeia 11.6 - General chapter 5.31 - Phage therapy medicinal products (France: Council of Europe). Available online at: www.edqm.eu.

[B9] Faruk O. Jewel Z. A. Bairagi S. Rasheduzzaman M. Bagchi H. Tuha A. S. M. . (2025). Phage treatment of multidrug-resistant bacterial infections in humans, animals, and plants: The current status and future prospects. Infect. Med. 4 (1), 100168. doi: 10.1016/j.imj.2025.100168, PMID: 40104270 PMC11919290

[B10] Garvey M. (2020). Bacteriophages and the one health approach to combat multidrug resistance: is this the way? Antibiotics 9, 414. doi: 10.3390/ANTIBIOTICS9070414, PMID: 32708627 PMC7400126

[B11] Gholizadeh O. Ghaleh H. E. G. Tat M. Ranjbar R. Dorostkar R. (2024). The potential use of bacteriophages as antibacterial agents against Klebsiella pneumoniae. Virol. J. 21, 191. doi: 10.1186/S12985-024-02450-7, PMID: 39160541 PMC11334591

[B12] Green S. (2024). Phage therapy is back, it’s promising and it’s going to take structural support to keep it going ( Springer Nature: Research Communities). Available online at: https://communities.springernature.com/posts/phage-therapy-is-back-it-s-promising-and-it-s-going-to-take-structural-support-to-keep-it-going.

[B13] Jansz N. Faulkner G. J. (2024). Viral genome sequencing methods: benefits and pitfalls of current approaches. Biochem. Soc. Trans. 52 (3), 1431–1447. doi: 10.1042/BST20231322, PMID: 38747720 PMC11346438

[B14] Jo S. J. Kwon J. Kim S. G. Lee S. J. (2023). The biotechnological application of bacteriophages: what to do and where to go in the middle of the post-antibiotic era. Microorganisms 11 (9), 2311. doi: 10.3390/microorganisms11092311, PMID: 37764155 PMC10534921

[B15] Jones J. D. Trippett C. Suleman M. Clokie M. R. J. Clark J. R. (2023). The future of clinical phage therapy in the United Kingdom. Viruses 15, 721. doi: 10.3390/V15030721, PMID: 36992430 PMC10053292

[B16] Kaur G. Agarwal R. Sharma R. K. (2021). Bacteriophage therapy for critical and high-priority antibiotic-resistant bacteria and phage cocktail-antibiotic formulation perspective. Food Environ. Virol. 13 (4), 433–446. doi: 10.1007/s12560-021-09483-z, PMID: 34120319

[B17] Lin R. C. Sacher J. C. Ceyssens P. J. Zheng J. Khalid A. Iredell J. R. (2021). Phage biobank: present challenges and future perspectives. Curr. Opin. Biotechnol. 68, 221–230. doi: 10.1016/J.COPBIO.2020.12.018, PMID: 33581425

[B18] Luong T. Salabarria A. C. Edwards R. A. Roach D. R. (2020). Standardized bacteriophage purification for personalized phage therapy. Nat. Protoc. 15, 2867–2890. doi: 10.1038/S41596-020-0346-0, PMID: 32709990

[B19] Marino A. Stracquadanio S. Cosentino F. Maraolo A. E. Colpani A. De Vito A. . (2025). Phage to ESKAPE: personalizing therapy for MDR infections—A comprehensive clinical review. Pathogens 14 (10), 1011. doi: 10.3390/pathogens14101011, PMID: 41156622 PMC12566643

[B20] Mayrhofer M. T. (2025). How the world of biobanking is changing with artificial intelligence. Front. Digit Health 7. doi: 10.3389/FDGTH.2025.1626833/BIBTEX, PMID: 40970141 PMC12440935

[B21] Mora-Domínguez J. Calero-Cáceres W. (2025). Addressing methodological bias in phageome research to clarify bacteriophage diversity. Lancet Microbe, 101285. doi: 10.1016/j.lanmic.2025.101285, PMID: 41205621

[B22] Mutalik V. K. Arkin A. P. (2022). A phage foundry framework to systematically develop viral countermeasures to combat antibiotic-resistant bacterial pathogens. iScience 25 (4), 104121. doi: 10.1016/j.isci.2022.104121, PMID: 35402883 PMC8983348

[B23] Niazi S. K. (2025). Bacteriophage therapy: discovery, development, and FDA approval pathways. Pharmaceuticals 18, 1115. doi: 10.3390/PH18081115, PMID: 40872508 PMC12389134

[B24] Nie W. Qiu T. Wei Y. Ding H. Guo Z. Qiu J. (2024). Advances in phage-host interaction prediction: in silico method enhances the development of phage therapies. Brief Bioinform. 25 (3), bbae117. doi: 10.1093/BIB/BBAE117, PMID: 38555471 PMC10981677

[B25] Panagiotopoulos A. P. Sagona A. P. Tsakri D. Ferous S. Anastassopoulou C. Tsakris A. (2025). Virological and pharmaceutical properties of clinically relevant phages. Antibiotics 14 (5), 487. doi: 10.3390/antibiotics14050487, PMID: 40426553 PMC12108485

[B26] Peng H. Chen I. A. Qimron U. (2025). Engineering phages to fight multidrug-resistant bacteria. Chem. Rev. 125 (2), 933–971. doi: 10.1021/acs.chemrev.4c00681, PMID: 39680919 PMC11758799

[B27] Resch G. Brives C. Debarbieux L. Hodges F. E. Kirchhelle C. Laurent F. . (2024). Between centralization and fragmentation: the past, present, and future of phage collections. PHAGE: Therapy Applications Res. 5 (1), 22–29. doi: 10.1089/phage.2023.0043, PMID: 40114810 PMC11920704

[B28] Samson R. Dharne M. Khairnar K. (2024). Bacteriophages: Status quo and emerging trends toward one health approach. Sci. Total Environ. 908, 168461. doi: 10.1016/J.SCITOTENV.2023.168461, PMID: 37967634

[B29] Sati H. Carrara E. Savoldi A. Hansen P. Garlasco J. Campagnaro E. . (2025). The WHO Bacterial Priority Pathogens List 2024: a prioritisation study to guide research, development, and public health strategies against antimicrobial resistance. Lancet Infect. Dis. 25, 1033–1043. doi: 10.1016/S1473-3099(25)00118-5, PMID: 40245910 PMC12367593

[B30] Strathdee S. A. Hatfull G. F. Mutalik V. K. Schooley R. T. (2023). Phage therapy: From biological mechanisms to future directions. Cell 186, 17–31. doi: 10.1016/J.CELL.2022.11.017, PMID: 36608652 PMC9827498

[B31] Turner D. Adriaenssens E. M. Tolstoy I. Kropinski A. M. (2021). Phage annotation guide: guidelines for assembly and high-quality annotation. PHAGE: Therapy Applications Res. 2 (4), 170–182. doi: 10.1089/phage.2021.0013, PMID: 35083439 PMC8785237

[B32] Wu P. Li W. Zhang W. Li S. Deng B. Xu S. . (2025). Advanced strategies in phage research: innovations, applications, and challenges. Microorganisms 13, 1960. doi: 10.3390/MICROORGANISMS13081960, PMID: 40871464 PMC12388022

[B33] Xing B. Liu C. Chen W. Li Z. Jing X. Wu C. . (2025). Gut Phage Biobank: a collection of bacteriophages targeting human commensal bacteria. Nat. Commun. 16, 11050. doi: 10.1038/s41467-025-61946-0, PMID: 41381457 PMC12698836

[B34] Zalewska-Piątek B. (2023). Phage therapy—Challenges, opportunities and future prospects. Pharmaceuticals 16 (12), 1638. doi: 10.3390/ph16121638, PMID: 38139765 PMC10747886

[B35] Zhao Y. Shu M. Zhang L. Zhong C. Liao N. Wu G. (2024). Phage-driven coevolution reveals trade-off between antibiotic and phage resistance in *Salmonella anatum*. ISME Commun. 4 (1), ycae039. doi: 10.1093/ismeco/ycae039, PMID: 38616926 PMC11014889

