# Correction: Phage biobanks as enabling infrastructure for precision phage therapy in the era of antimicrobial resistance

**DOI:** 10.3389/frabi.2026.1804100

**Published:** 2026-02-11

**Authors:** Paulina Topa-Pila, Katheryne Y. Morales, Doménica P. Palacios-Mora, Cristina Flores-Hernández, Analía Altamirano-Cisneros, Joselyn Micaela Gavilanes, Keyla Villacís-López, Mirari Arancibia, Johanna Mora-Domínguez, William Calero-Cáceres

**Affiliations:** 1 Biotechnology Program, Department of Food and Biotechnology Science and Engineering, Universidad Técnica de Ambato, Ambato, Ecuador; 2 Clínica Santa Ana, Cuenca, Ecuador; 3 Departamento de Ciencias Exactas Sede Latacunga, Universidad de las Fuerzas Armadas ESPE, Rumiñahui, Ecuador; 4 Hospital de Especialidades José Carrasco Arteaga, Instituto Ecuatoriano de Seguridad Social (IESS), Cuenca, Ecuador; 5 UTA-RAM-One Health, Group for Universal Advance in Bioscience, Department of Food and Biotechnology Science and Engineering, Universidad Técnica de Ambato, Ambato, Ecuador

**Keywords:** antimicrobial resistance (AMR), bioinformatics, One health, phage biobanks, phage therapy, precision medicine

Author Analía Altamirano-Cisneros was erroneously spelled as AnalÃŁa Altamirano. The **Author contributions** statement, citation and copyright have been corrected accordingly.

The original version of this article has been updated.

